# Chloroquine suppresses proliferation and invasion and induces apoptosis of osteosarcoma cells associated with inhibition of phosphorylation of STAT3

**DOI:** 10.18632/aging.203196

**Published:** 2021-06-24

**Authors:** Chenglong Chen, Hongliang Zhang, Yiyang Yu, Qingshan Huang, Wei Wang, Jianfang Niu, Jingbing Lou, Tingting Ren, Yi Huang, Wei Guo

**Affiliations:** 1 Musculoskeletal Tumor Center, Peking University People’s Hospital, Beijing, People’s Republic of China; 2 Beijing Key Laboratory of Musculoskeletal Tumor, Beijing, People’s Republic of China

**Keywords:** chloroquine, osteosarcoma, apoptosis, metastasis, p-STAT3

## Abstract

Background: Osteosarcoma (OS) is characterized by a high rate of metastasis. It has been found that tumor cells can bypass apoptosis which leads to an uncontrolled proliferation, but chloroquine (CQ) can have an effect on the tumors by inducing apoptosis. We aimed to explore the effects and the hypothetical mechanism of CQ effects on OS.

Methods: We first estimated the CQ effects on proliferation, apoptosis, migration, invasion, and lamellipodia formation of OS cells. Mice bearing xenograft model were used to test the anti-tumor growth and lung metastasis effects of CQ in OS. Western blot and immunohistochemistry were used to explore the mechanism of CQ effects and the association between p-STAT3 expression and lung metastasis of OS patients.

Results: CQ induces the apoptosis and suppressed the viability, proliferation, migration, invasion, and lamellipodia formation of OS cells *in vitro*. *In vivo* experiments demonstrated that CQ inhibited tumor growth and lung metastasis. CQ induced apoptosis was dependent on the lysosomal inhibition and inhibition of protein turnover. The lung metastasis was associated with the p-STAT3 expression in OS patients.

Conclusion: CQ inhibited progression of OS cells *in vitro*, and suppressed tumor growth and lung metastasis *in vivo*. p-STAT3 can be a predictive biomarker for lung metastasis in osteosarcoma patients.

## INTRODUCTION

Osteosarcoma (OS) is the most common malignant bone tumor in teenagers, and has a high propensity for pulmonary metastasis [1]. Currently, the standard care for OS consists of extensive surgical resection, neoadjuvant chemotherapy, and adjuvant chemotherapy [2]. Although numerous novel anticarcinogens have been clinically applied, including immune checkpoint inhibitors and molecular-targeting drugs, the prognosis for recurrent and metastatic OS has remained stagnant over the last decades owing to the poor response rate to these drugs [3]. Given these barriers to current regimens, there is an urgent need to establish novel therapeutic strategies that may improve the overall survival of OS. Immunotherapy, a novel anti-cancer approach showed no apparent therapeutic effects on solid tumors including OS. This may be because the microenvironment of OS is a barren field of T lymphocytes (T cells) and most immunotherapies utilize a T cell-based approach. While focusing on T cell-based immunotherapy, more attention needs to be paid to drugs acting on tumor cells and the relevant signaling pathways.

Apoptosis is a caspase-dependent form of programmed cell death that plays a crucial role in organism development and tissue homeostasis under normal conditions [4]. However, cancer cells can bypass this suicidal process, leading to uncontrolled proliferation through overexpression of some proteins to activate or inhibit apoptosis signaling pathways. Studies have reported that many approaches inducing apoptosis showed encouraging effects in treating cancers such as melanoma [5], lung cancer [6], ovarian cancer [7], and glioblastoma [4].

Chloroquine (CQ) has long been used to treat malaria and has been reported to affect tumors through the induction of apoptosis [5, 8, 9]. Although CQ is generally used as an inhibitor of autophagy, depending on the type of cancer, autophagy may become dysregulated in a way that enables tumor cell survival even in a nutritionally limited environment [5, 10, 11]. The effects and mechanisms of action of CQ on OS remain unclear. Bcl-2, an important inhibitor of apoptosis proteins, can inhibit cell death upstream of the mitochondria before caspase activation [12], and STAT3 is one of the upstream proteins directly regulating bcl-2. Studies have indicated that activating the p-STAT3/bcl-2 signaling pathway suppresses apoptosis in several cancers [7, 13, 14]. Induction of apoptosis may inhibit OS by regulating the related signaling pathways. Here, we used CQ to reveal the effects of p-STAT3/bcl-2/Caspase3 signaling-associated apoptosis in OS cells and xenograft models, and to predict prognosis by testing p-STAT3 expression in OS patients.

## RESULTS

### CQ induces the apoptosis and inhibits the proliferation of OS cells

**Figure 1:**
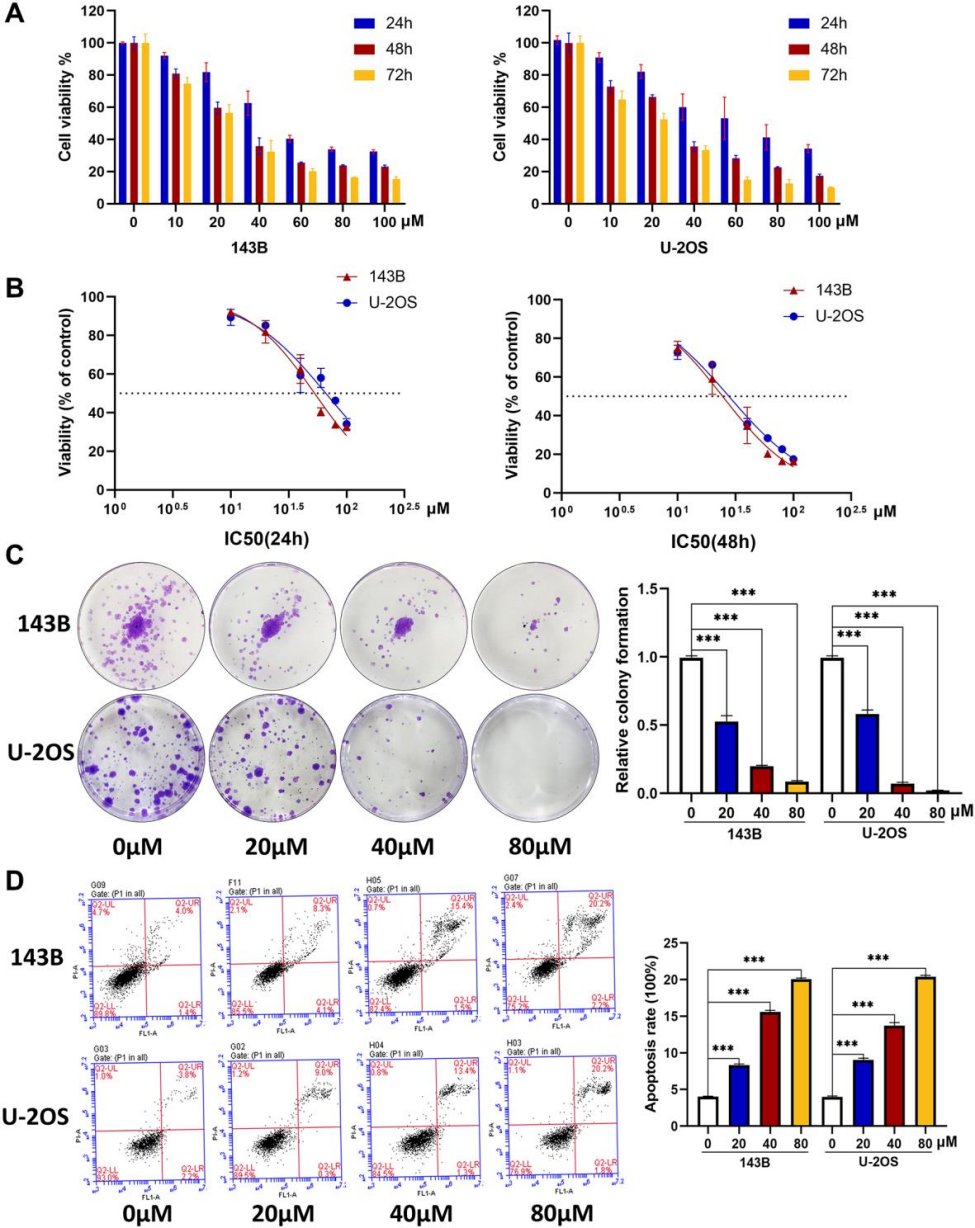
**CQ induces the apoptosis and inhibits the viability and proliferation of OS cells.** (**A**) The OS cell viability at 24 h, 48 h, and 72 h after treated with CQ (*n* = 3). (**B**) The IC50 value at 24 h and 48 h in 143B and U-2OS cells (*n* = 3). (**C**) The colony formation assay showed that the proliferation of OS cells was inhibited gradually by CQ with the increasing of concentration (^***^p < 0.001; *n* = 3). (**D**) Flow cytometry showed that CQ could regulate apoptosis of OS cells (^***^p < 0.001; *n* = 3). All the data were expressed as the mean ± SD.

We first tested the cytotoxicity of CQ on OS cells using the CCk-8 assay, and the results showed cell viability at 24 h, 48 h, and 72 h in a concentration gradient of CQ (Figure 1A), and the IC50 values at 24 h and 48 h in 143B and U-2OS cells (Figure 1B). Specifically, the IC50 value in 143B at 24h and 48h were 53.06 μM and 24.54 μM respectively, and were 66.3 μM and 27.81 μM in U2OS, respectively. As illustrated in Figure 1C, the proliferation of OS cells was significantly inhibited by CQ with increasing concentration. Furthermore, the flow cytometry results showed that with increasing CQ concentration, more apoptosis occurred in OS cells (Figure 1D). These results indicated that CQ was cytotoxic to OS cells, inhibited their proliferation and induced apoptosis *in vitro* in a dose-and time-dependent manner.

### CQ suppresses the migration, invasion, and lamellipodia formation of OS cells

**Figure 2:**
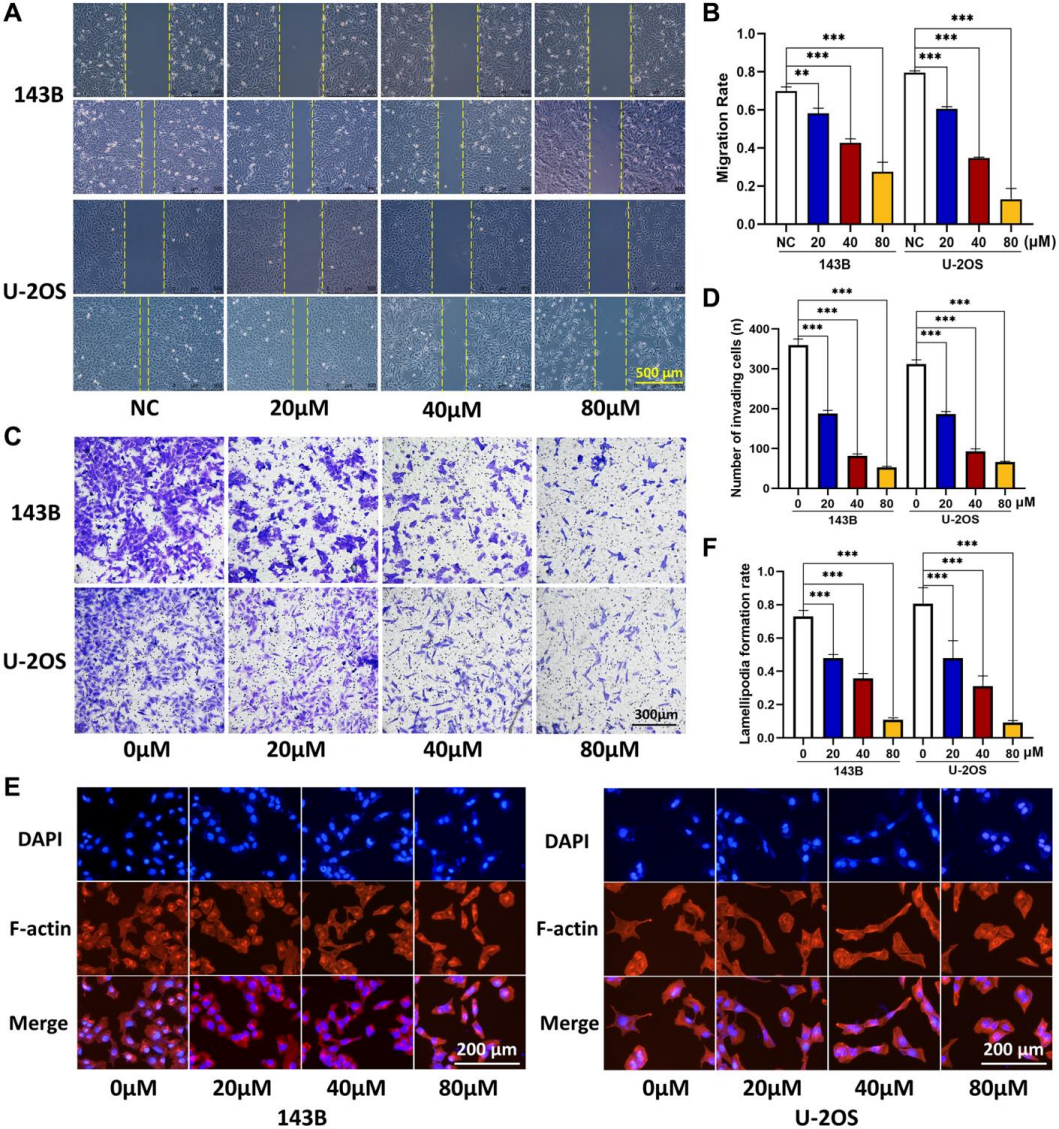
**CQ suppress the OS cell migration, invasion, and lamellipodia formation.** (**A**, **B**) Migration ability of OS cells was inhibited gradually by CQ with the increasing of concentration (^**^P < 0.01; ^***^p < 0.001; *n* = 3). (**C**, **D**) Invasion ability of OS cells was inhibited gradually by CQ with the increasing of concentration (^***^p < 0.001; *n* = 3). (**E**, **F**) Cytoskeletal assay showed that the lamellipodia formation of OS cells was inhibited gradually by CQ with the increasing of concentration (^***^p < 0.001; *n* = 3). The data were expressed as the mean ± SEM.

To further examine the effects of CQ on the function of OS cells *in vitro*, we conducted wound healing, transwell invasion, and cytoskeletal assays. As shown in Figure 2A–2D, the results of wound-healing and transwell invasion assays showed that CQ significantly inhibited the migration and invasion ability of both 143B and U-2OS cells. In the cytoskeletal assay, the results showed that a reconstructed cytoskeleton, well-distributed F-actin, and fewer lamellipodia were observed in the CQ-treated group compared with those in the NC group (Figure 2E, 2F).

### CQ inhibits tumor growth and lung metastasis *in vivo*

**Figure 3:**
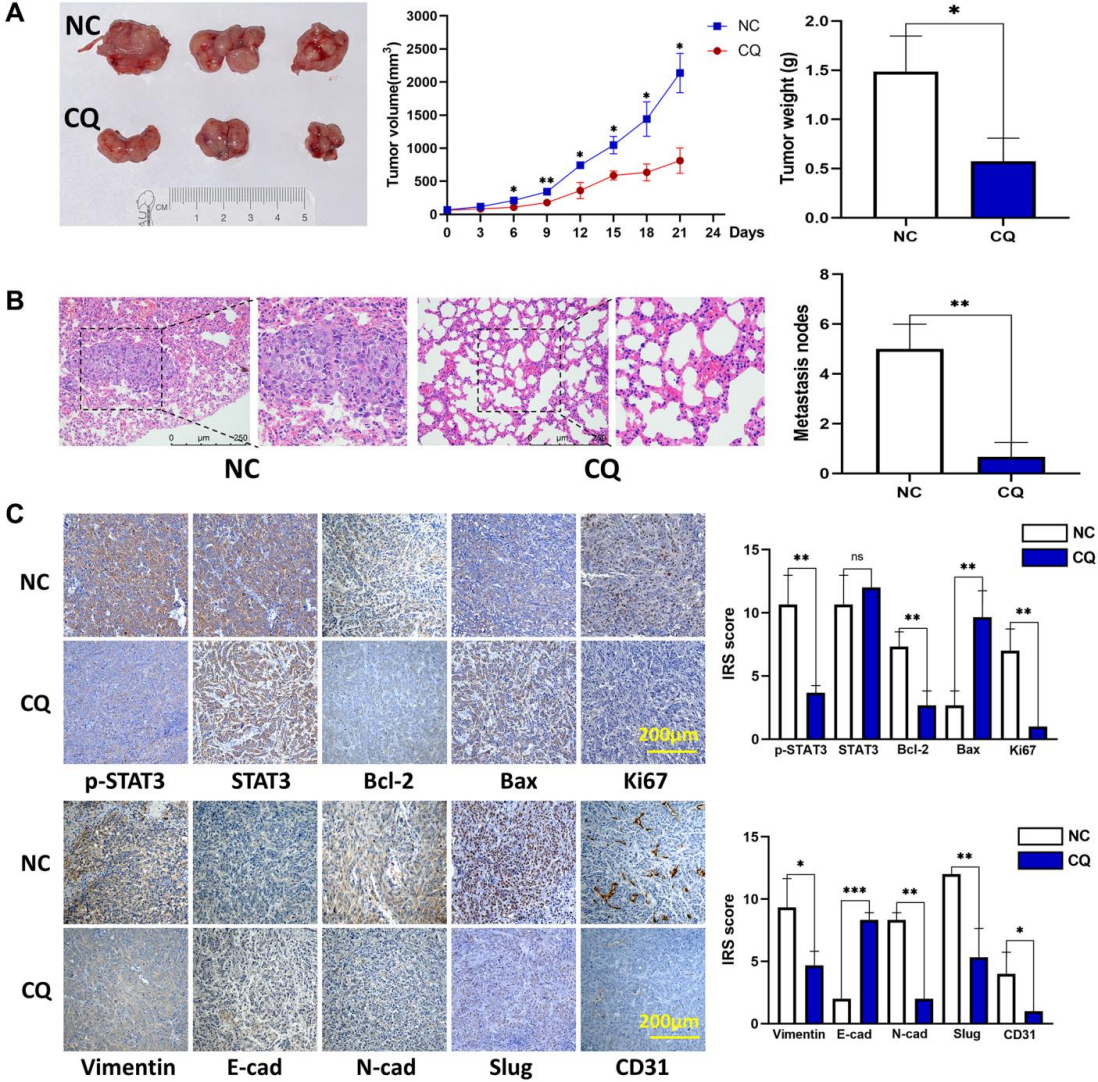
**CQ suppression of mouse OS cell xenograft growth and lung metastasis *in vivo*.** (**A**) Xenograft photographs, growth curves of tumor, and tumor weight showed that CQ suppressed xenograft growth in nude mice (^*^p < 0.05; *n* = 3). (**B**) HE staining of mouse lung tissues. The results showed suppression of lung metastasis *in vivo* (^**^p < 0.01; *n* = 3). (**C**) Representative pictures from IHC assay. p-STAT3, STAT3, Bcl-2, Bax, Ki67, Vimentin, E-cadherin, N-cadherin, Slug, and CD31 expression in the tumors collected from different groups were determined using IHC (ns *p* > 0.05; ^*^p < 0.05; ^**^p < 0.01; ^***^p < 0.001; *n* = 3). The data were expressed as the mean ± SD.

*In vivo* experiments with a xenograft model indicated that after treatment with CQ for 3 weeks, the tumor growth rate and tumor weight were significantly reduced (Figure 3A). Furthermore, we calculated the number of lung metastasis nodules in each group and found that fewer nodules were observed in the CQ group than those in the NC group (Figure 3B). IHC results showed that the expression of these proteins associated with tumor progression was affected by CQ (Figure 3C). These results verified the anticancer effects of CQ *in vivo*.

### CQ regulates the apoptosis, proliferation, migration, and invasion of OS cells by inhibiting the phosphorylation of STAT3

**Figure 4:**
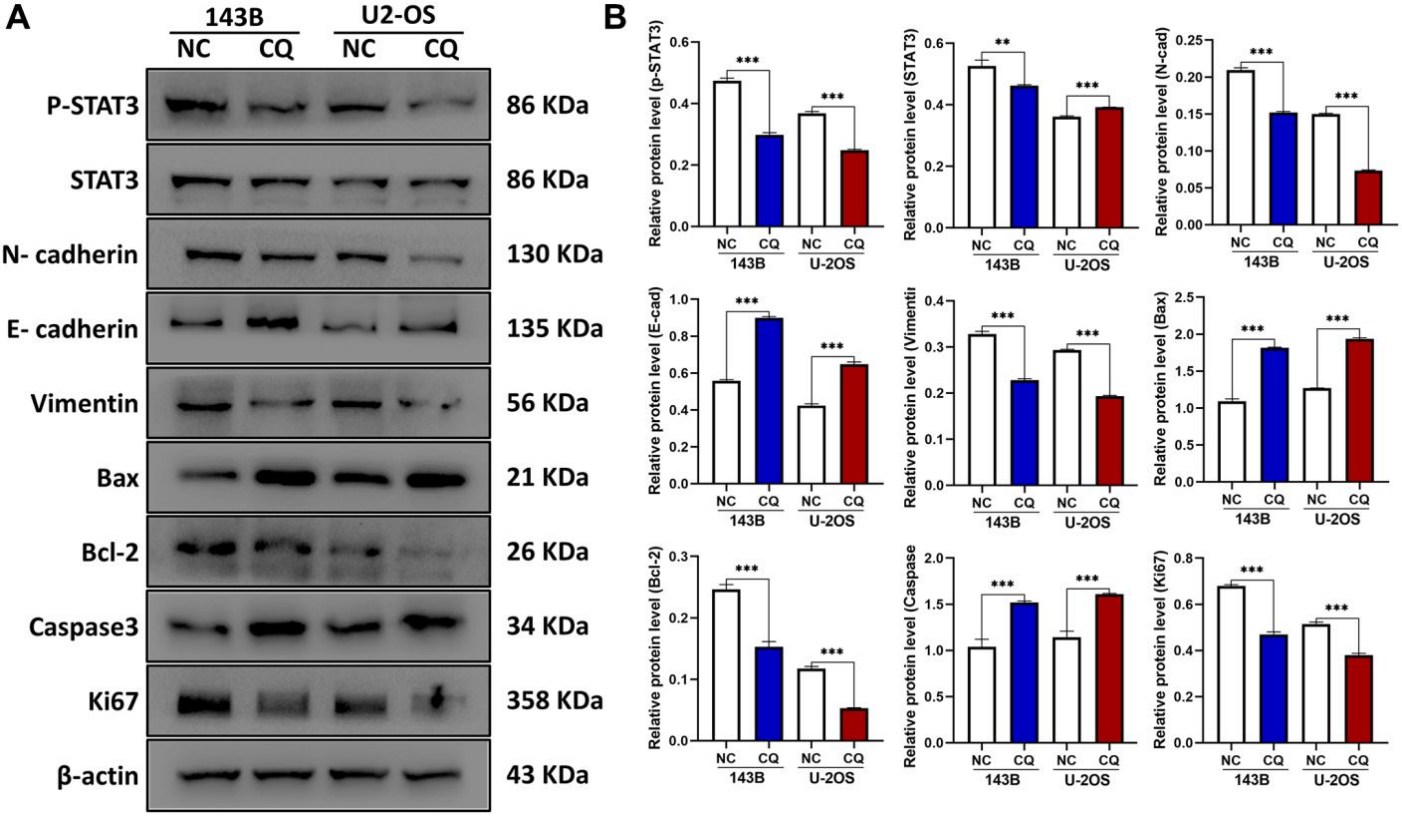
**The expression of E-cadherin, Bax, Caspase3, and CD31 were up-regulated, and p-STAT3, N-cadherin, Vimentin, Bcl-2, Ki67, Slug were downregulated by CQ treatment in OS cells.** (**A**) Representative pictures from tumor cell western blot assay. (**B**) The graph is the quantified data of the western blot, and β-actin was used as a load control (^**^p < 0.01; ^***^p < 0.001 *n* = 3). The data were expressed as the mean ± SD.

STAT3 is a well-known tumor therapeutic target, and our previous studies have demonstrated that STAT3 is also highly expressed in OS and showed apoptosis-associated effects [2, 15, 16], We investigated whether the STAT3 pathway is involved in CQ action in OS cells and xenograft tumor samples. Western blot results demonstrated that the expression of p-STAT3 and its target bcl-2 was reduced after treatment with CQ in OS cells (Figure 4A, 4B). The expression of proteins related to proliferation (ki67), apoptosis (Bax/bcl2/Caspase3), epithelial-mesenchymal transition (EMT) and metastasis (N-cadherin/ E-cadherin), and cytoskeleton formation (vimentin) were detected (Figure 4A, 4B). These results are consistent with those of our cell function experiments. Moreover, IHC results further confirmed the effects of CQ on the expression of proteins related to proliferation, apoptosis, cytoskeleton, EMT and metastasis (SLUG), and angiogenesis (CD31) *in vivo* (Figure 3C).

### Positive p-STAT3 expression is significantly associated with OS grade and distant metastasis, and it predicted a short overall survival time

**Figure 5:**
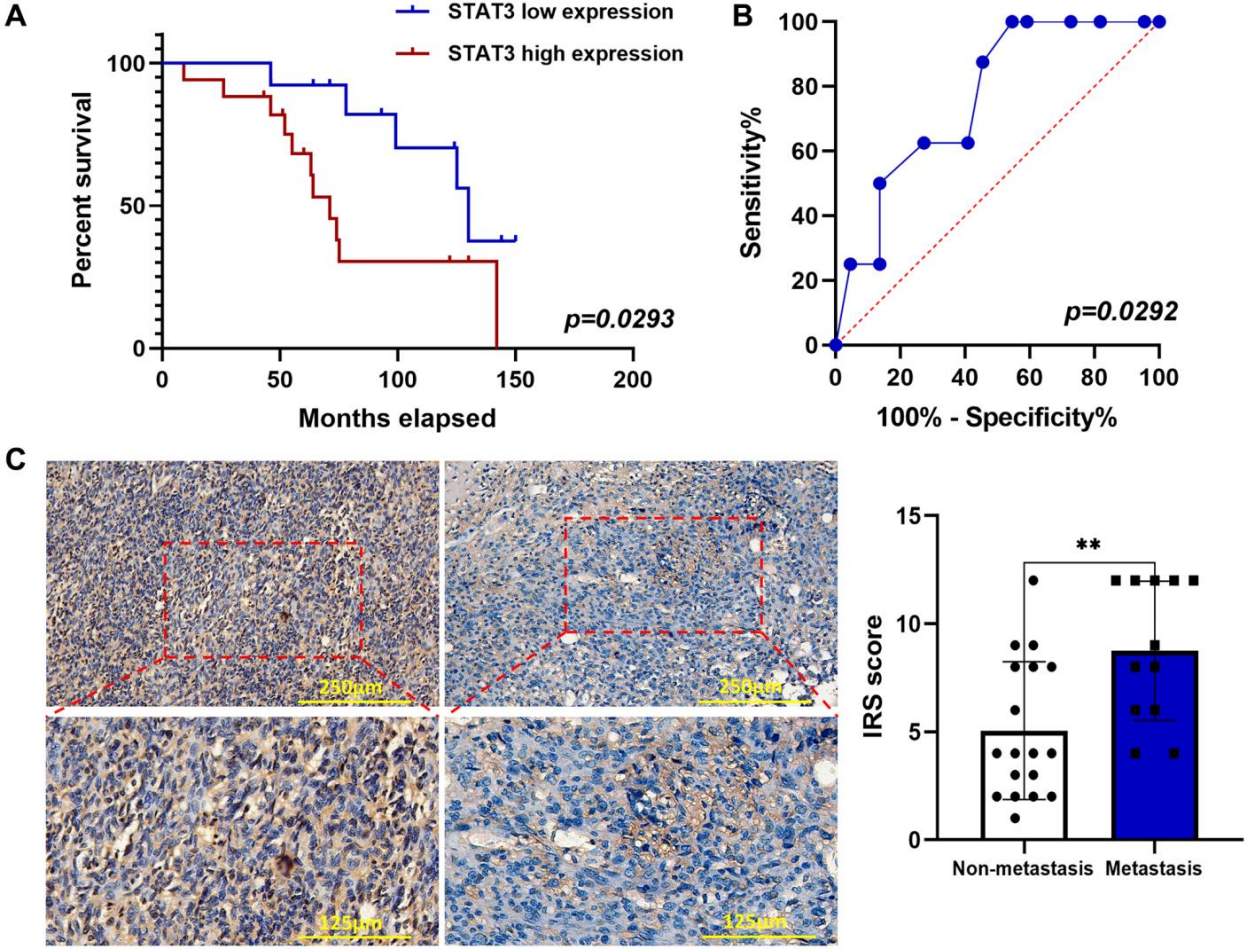
**p-STAT3 expression was elevated by IHC in OS and associated with poor prognosis.** (**A**) Kaplan–Meier curves showed the p-STAT3 expression on overall survival in 30 OS patients (^*^p < 0.05; *n* = 30). (**B**) ROC curve of predictive value of p-STAT3 expression on lung metastasis of OS patients (^*^p < 0.05; *n* = 30). (**C**) IHC staining of p-STAT3 in OS samples (^**^p < 0.01; *n* = 30). The data were expressed as the mean ± SD.

To further verify whether p-STAT3 expression was associated with the prognosis of OS patients, 30 OS tissue samples were assessed using IHC (Table 1). We found that the positive rate of p-STAT3 was higher in patients with poor prognosis than that in patients with better outcomes (Figure 5A). The specific performance was high pulmonary metastasis rate and shorter overall survival time. We used ROC curves to further evaluate whether p-STAT3 might be a predictive biomarker for lung metastasis, and the results showed that the area under the ROC curve for p-STAT3 was 0.7642 (Figure 5B). Lung metastasis was associated with p-STAT3 expression in patients with OS (Figure 5C). This indicates that p-STAT3 expression is valuable in predicting OS lung metastasis.

**Table 1:** Association between p-STAT3 expression and clinical characteristics of OS patients.

**Variables**	**Cases**	**p-STAT3 Lower Expression**		**p-STAT3 Higher Expression**		***P*-value**
		**N**	**%**	**N**	**%**	
**Gender**						0.999
Male	17	7	53.8	10	58.8	
Female	13	6	46.2	7	41.2	
**Age at diagnosis (years)**						0.443
≤20	21	8	61.5	13	76.5	
>20	9	5	38.5	4	23.5	
Tumor location						0.920
Femur	11	4	30.8	7	41.2	
Tibia	8	4	30.8	4	23.6	
Humerus	6	3	23.1	3	17.6	
Others	5	2	15.3	3	17.6	
**Histological types**						0.543
Osteoblastic	15	7	53.8	8	47.1	
Chondroblastic	12	4	30.8	8	47.1	
Others	3	2	15.4	1	5.8	
**Lung metastasis occurs at the time of diagnosis**						0.026^*^
Occurred	12	2	15.4	10	58.8	
Not occurred	18	11	84.6	7	41.2	

Notes: χ^2^ (fisher’s exact) test, ^*^p < 0.05.

### The apoptosis is dependent on the inhibition of protein turnover and lysosome, and enhancement of protein stability

**Figure 6:**
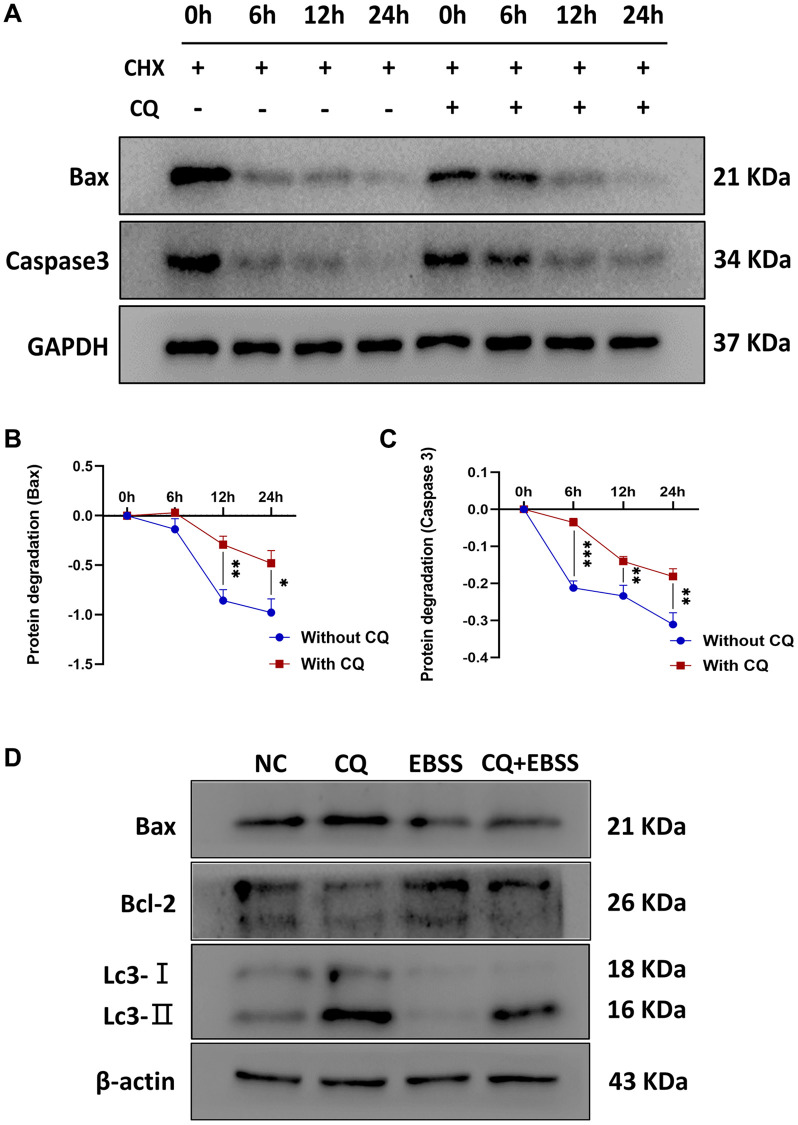
**CQ reduced the protein degradation of Bax and Caspase3, and CQ-induced apoptosis of OS cells was dependent on the lysosomal inhibition.** The (**A**) Representative pictures from tumor cell western blot assay. (**B**) The graph is the quantified data of the western blot for the expression of BAX, and GAPDH was used as a load control (^*^p < 0.05; ^**^p < 0.01; *n* = 3). (**C**) The graph is the quantified data of the western blot for the expression of Caspase3, and GAPDH was used as a load control (^**^p < 0.01; ^***^p < 0.001; *n* = 3). (**D**) Representative pictures from 143B cells western blot assay and the cells were treated with PBS, 20 μM CQ, EBSS, or the combination of CQ and EBSS. The data were expressed as the mean ± SD.

**Figure 7:**
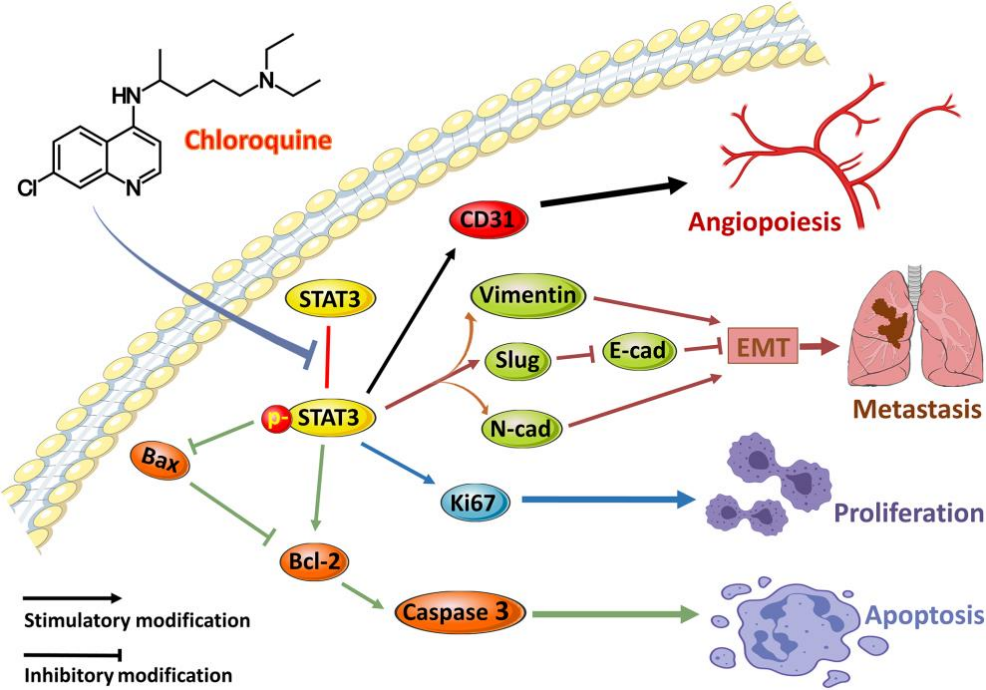
**The schematic of the hypothetic mechanisms of CQ effects on p-STAT3/Bcl-2/Caspase3 pathway.** CQ inhibited the phosphorylation of STAT3 and then by downregulating the expression of p-STAT3 to suppress the CD31 to inhibit angiopoiesis, and suppress N-cadherin, Vimentin, and Slug as well as downregulate E-cadherin to inhibit EMT and metastasis, and suppress Ki67 to inhibit proliferation, as well as upregulate Bax and Caspase3 and downregulate Bcl-2 to induce apoptosis of OS cells.

To verify the hypothesis that lysosomal inhibition is responsible for the apoptosis of OS cells, 143B cells were treated with PBS, 20 μM CQ, Earle's balanced salt solution (EBSS), or the combination of CQ and EBSS for protein expression detection of Bax, Bcl-2, and Lc3. The results showed Bax and Caspase3 degradation were reduced by CQ, indicating that Bax and Caspase3 induction was partly the result of the inhibition of protein turnover by CQ after treatment with cycloheximide which inhibits the protein synthesis in osteosarcoma cells (Figure 6A–6C); and CQ induced apoptosis is related to lysosomal inhibition, or at least as a cause, in OS cells (Figure 6D). Figure 7 showed the schematic of the hypothetic mechanisms of CQ effects on the STAT3 pathway and apoptosis.

## DISCUSSION

Pulmonary metastasis is a major obstacle that limits OS patient survival [17]. Although several anticarcinogens, especially chemotherapeutic drugs, have been clinically applied, the prognosis of OS has remained stagnant over the last few decades [3]. The main reasons for the failure of chemotherapy drugs are the emergence of chemotherapy resistance and serious side effects. Therefore, novel treatment approaches are required for patients with OS. In this study, we found robust antineoplastic effects of CQ monotherapy in OS cells and xenograft models for the inhibition of cell migration, invasion, and lung metastasis. We further elucidated the mechanisms underlying CQ-induced apoptosis in OS cells. This may provide new insights and a theoretical basis for the future use of CQ in the treatment of OS.

CQ has been reported to activate multiple processes and has been used to treat different types of cancers. In this study, we focused on the apoptosis-inducing effects of CQ in suppressing OS *in vitro* and *in vivo*. We first tested the cytotoxicity of CQ to OS cells using CCk-8 and confirmed our hypothesis that CQ could have inhibitory effects on both 143B and U-2OS cells. The flow cytometry results showed that CQ induced the apoptosis of OS cells gradually with increasing concentration. Next, we examined the proliferation ability of OS cells after treatment with different concentrations of CQ, and the results indicated that CQ inhibited cell proliferation in a concentration-dependent manner. Our results were in accordance with those of a previous study of CQ in the suppression of pituitary tumor cell proliferation [18]. The western blot results for detecting Ki67 expression, which are generally used as a proliferation indicator, were consistent with the clone formation assay and indicated that CQ reduced Ki67 expression to inhibit the proliferation of OS cells.

Bax, a proapoptotic protein, is a member of the Bcl-2 family which can accelerate the process of apoptosis by binding directly to Bcl-2 to form a dimer [19]. In the final phase of apoptosis, essential cell structures are dismantled by activation of downstream effector caspases, especially Caspase-3 [20]. However, this process can be blocked by anti-apoptotic proteins such as Bcl-2. Studies have reported that Bcl-2 is highly expressed in several cancers and can protect tumor cells from apoptosis by inhibiting the adapters required for activation of the caspases that dismantle the cells [21]. Our western blot results elucidated the mechanism underlying CQ-induced apoptosis in OS, which reduced Bcl-2 expression by inhibiting its upstream pathway protein p-STAT3, and then released the inhibition of Bax and Caspase-3. These alterations in the expression of proteins eventually induce and promote apoptosis in OS. Similarly, these abilities and functions such as EMT and metastasis, which are vital to tumor cells, are determined by the expression of related proteins. We assessed the migration, invasion, and lamellipodia formation abilities after treatment with different concentrations of CQ in OS, and further analyzed the expression of N-cadherin, E-cadherin, and vimentin. The results indicated that by suppressing the expression of these proteins, CQ restrained the migration, invasion, and lamellipodia formation in OS cells, which may provide a theoretical foundation for the use of CQ in OS.

To further examine the anti-tumor effects of CQ *in vivo*, we conducted experiments using a xenograft tumor model. Based on our results, CQ proved to be an anticarcinogen in inhibiting tumor growth and lung metastasis in tumor-bearing mice. Our IHC results verified the *in vitro* experiment of western blot and further confirmed that CQ reduced the expression of SLUG and CD31 in xenograft tumor. This indicated that in addition to inhibiting EMT and metastasis, CQ may serve as an antiangiogenic drug by suppressing CD31. To verify our hypothesis that p-STAT3 is the key for CQ-induced apoptosis and can serve as a prognostic indicator in OS patients, we tested p-STAT3 expression in tissues from OS patients and evaluated their prognosis. Our data demonstrated that OS patients tend to have poorer prognosis and higher lung metastasis rates with increased p-STAT3 expression. This provided new evidence for the use of p-STAT3 as a predictive target for lung metastasis in patients with OS. It is also well established in other cancers that p-STAT3 can play crucial roles in inducing cancer cell proliferation, tumor immunosuppression, angiogenesis, anti-apoptosis, and metastasis [22, 23, 24, 25]. Studies have indicated that JAK–STAT3 signalling pathway significantly contribute to the formation of pre-metastatic niches in future cancer metastatic sites. Figure 7 shows the hypothetical mechanism by which CQ regulates the expression of multiple proteins, especially STAT3 phosphorylation, and ultimately affects the progression of OS. Although the mechanism of STAT3 in oncogenesis and progression has not been fully elucidated, according to our results, the inhibitory effects of CQ on p-STAT3 is a potential target in OS. In this study we further demonstrate that CQ-induced apoptosis is dependent on the lysosomal inhibition. Our results indicated that the BAX induction is at least in part the result of inhibition of protein turnover by CQ, and CQ can enhance the protein stability of Caspase-3. The results suggest that the effect on p-STAT expression or the STAT signalling by CQ is related with autophagy, and this is accord with previous studies [26].

In conclusion, our research demonstrates that CQ exerts antitumor effects on OS cells both *in vitro* and *in vivo*. The benefits can be interpreted by p-STAT3-mediated Bcl-2 deactivation and the increase of Bax and Caspase-3, and the expression changes of targets inhibiting proliferation and EMT. Meanwhile, the inhibition of autophagy was also responsible for the apoptosis of OS cells, and this was accord with the previous studies that autophagy as a protection factor in OS [26, 27]. This study highlights the potential of p-STAT3 as a new therapeutic target that can be inhibited by CQ in OS. Our study had some limitations. This is a small sample size study that lacks comprehensive investigation of CQ-induced apoptosis downstream signalling and an in-depth scientific rationale. Similarly, our proposed mechanisms based on different doses of CQ in *in vitro* and *in vivo* study for exerting anti-tumor effects need to be further investigated. We expect that our future studies will further validate the therapeutic efficacy of CQ and our hypothetical mechanism and provide better insights into the role of apoptosis inhibition in the treatment of OS.

## MATERIALS AND METHODS

### Cell lines and cell culture

Human osteosarcoma 143B and U-2OS cell lines (ATCC, American Type Culture Collection, Manassas, VA, USA) were maintained in Dulbecco’s modified Eagle’s medium (DMEM, HyClone) and RPMI-1640 medium (Gibco) respectively. All growth media were supplemented with 10% fetal bovine serum (FBS, Gibco) and 1% penicillin/streptomycin (Gibco), and all cells were cultured at 37°C in a humidified incubator with 5% CO^2^.

### Cell viability, colony formation, and apoptosis assays

We used the CCk-8 assay (Dojindo, Japan) to estimate the cytotoxicity of CQ on OS cells. 143B and U-2OS cells were seeded in 96-well plates at 1 × 10^3^ per well overnight and then treated with different concentrations of CQ for 24 h, 48 h, and 72 h. To determine the effect of CQ on the proliferation of OS cells, 1 × 10^3^ 143B or U-2OS cells were seeded in 6-well plates, and then treated with CQ at 0 μM, 20 μM, 40 μM, or 80 μM for 12 h. The cells were fixed with 4% paraformaldehyde and stained with 0.5% crystal violet for 15 min. Apoptosis of OS cells was analyzed by flow cytometry. The cells were treated with CQ at the mentioned concentrations for 24 h and then examined by flow cytometry after staining with FITC Annexin V apoptosis detection kit (BD Biosciences).

### Wound-healing, transwell invasion, and cytoskeletal assays

For the wound-healing assay, as we described previously [28], the cells were cultured to 80%–90% confluence in 6-well plates and then treated with different concentrations of CQ in serum-free medium. Then, an artificial cell-free area was wounded by scratching with a P-200 pipette tip. The wound area was photographed at 0 and 24 h under a phase-contrast microscope. For the transwell invasion assay, according to our previous studies [28, 29], 143B (3 × 10^4^) and U-2OS (5 × 10^4^) cells in serum-free medium with different concentrations of CQ were added into a Matrigel-coated 8-μm filter chamber, and the lower chamber was filled with 600 μL complete medium. After 24 h, the cells that migrated across the membrane were fixed with 4% paraformaldehyde and stained with 0.5% crystal violet. For the cytoskeletal assay, cells treated with different CQ concentrations were fixed with 4% paraformaldehyde when cultured to semi-confluence. The cells were then treated with 0.5% Triton X-100 (Solarbio, Beijing, China) for 5 min and stained with 100 nM rhodamine-phalloidin in the dark for 30 min. Cell nuclei were stained with 100 nM DAPI (Solarbio) for 1 min. The cell cytoskeleton and its fluorescence intensity were analyzed using a fluorescence microscope.

### Mice bearing xenograft models

Five-week-old BALB/c female nude mice (Vital River, Beijing, China) were divided randomly into two groups, and implanted subcutaneously in the flank region with 3 × 10^6^ 143B cells mixed with 100 μl Matrigel (Corning). After the tumors had grown, the mice were intraperitoneally injected with CQ (25 mg/kg) or normal saline once daily, and tumor growth was monitored using calipers every third day and calculated as length × (width)^2^ × 0.52. Subsequently, the mice were sacrificed for the harvesting of xenografts and lungs. All animal experiments were performed with written confirmation authorized by the Animal Care and Use Committee of Peking University People’s Hospital. Animal experiments complied with the ARRIVE guidelines and followed the National Institutes of Health Guide for the Care and Use of Laboratory Animals.

### Immunohistochemistry (IHC)

Paraffin sections of xenografts and tissue samples from OS patients were incubated with target antibodies (anti-STAT3, anti-p-STAT3, anti-N-cadherin, anti-E-cadherin, anti-Vimentin, anti-Bax, anti-Bcl-2, anti-SLUG, anti-CD31, and anti-ki67; Abcam) as described previously [15, 29]. The number of positive cells and the intensity of antibody staining were counted and determined under a microscope, and the data were evaluated using the immunoreactive score (IRS) system as previously described [29]. Written informed consent was obtained from all patients, and the study was approved by the Ethics Committee of Peking University People's Hospital.

### Hematoxylin and eosin (HE) staining

As described previously [29], mouse lung tissues were resected and sectioned at 100 μm intervals to eventually yield 10-15 sections per sample for HE staining. The tissue sections were then scanned using a Pannoramic MIDI digital slide scanner (3DHISTECH, Ltd. Budapest, Hungary), and the number of lung metastatic nodule was quantified in every section for statistical analysis.

### Western blot and reagents

Protein isolation of OS cells and western blot were performed as previously described [29, 30]. Protein samples from 143B and U-2OS cells treated with either CQ or PBS were analyzed for protein expression using specific antibodies. All protein quantification was normalized to β-actin as the internal reference. To explore the mechanism by which CQ affects the expression of Bax and Caspase3 in osteosarcoma cells. 143B cells were treated with 40 μM CQ or 20 μg/mL cycloheximide. The protein of cells was extracted at 0h, 6 h, 12 h, and 24 h, and western blot was subsequently performed. To verify our hypothesis that lysosomal inhibition is responsible for the apoptosis of OS cells, we treated 143B cells with PBS, 20 μM CQ, EBSS, or the combination of CQ and EBSS for 6 h, and then tested the Bax, Bcl-2, and Lc3 expression in each group. The following antibodies purchased from Abcam (USA) were used: anti-p-STAT3, anti-STAT3, anti-N-cadherin, anti-E-cadherin, anti-vimentin, anti-Bax, anti-bcl-2, anti-Caspase3, anti-LC3, and anti-ki67.

### Patients and clinicopathological characteristics

Thirty paraffin-embedded specimens of OS patients were obtained from the Department of Pathology at Peking University People's Hospital. None of the patients received preoperative radiotherapy, and no metastasis occurred at the time of diagnosis. Ethical approval was obtained from the ethics committee of Peking University People's Hospital and all the patients or their guardians provided written informed consent. The overall survival was defined as the interval between the first diagnosis and death, or the last follow-up. Outpatient visits or telephone calls were conducted to determine patient status. The primary lesion and chest X-ray examinations were performed every 3–6 months.

### Ethics statement

This study was approved by the ethics committee of Peking University People’s Hospital. Animal experiments complied with the ARRIVE guidelines and followed the National Institutes of Health Guide for the Care and Use of Laboratory Animals.

### Statistical analysis

GraphPad Prism 8 was used for statistical analyses. The data are presented as mean ± SD and statistical differences are indicated by *p* < 0.05. The differences between groups were analyzed by Student’s t-test or one-way ANOVA. We used the Kaplan–Meier method to estimate overall survival with the log-rank test. The association between the expression of p-STAT3 and the clinicopathological variables was assessed using the χ^2^ test. The predictive value of p-STAT3 expression in metastasis was assessed by a receiver-operating characteristic (ROC) curve. ^*^p < 0.05; ^**^p < 0.01; ^***^p < 0.001.
